# In vitro erythrocyte production using human-induced pluripotent stem cells: determining the best hematopoietic stem cell sources

**DOI:** 10.1186/s13287-023-03305-8

**Published:** 2023-04-26

**Authors:** Youn Keong Cho, Hyun-Kyung Kim, Soon Sung Kwon, Su-Hee Jeon, June-Won Cheong, Ki Taek Nam, Han-Soo Kim, Sinyoung Kim, Hyun Ok Kim

**Affiliations:** 1 Department of Laboratory Medicine, Yonsei University College of Medicine, Seoul, Republic of Korea; 2 Department of Internal Medicine, Yonsei University College of Medicine, Seoul, Republic of Korea; 3 Severance Biomedical Science Institute, Brain Korea 21 PLUS Project for Medical Science, Yonsei University College of Medicine, Seoul, Republic of Korea; 4 Department of Biomedical Sciences, Catholic Kwandong University College of Medical Convergence, Gangneung-si, Gangwon-do Republic of Korea

**Keywords:** Erythropoiesis, Hematopoietic stem cells, Human-induced pluripotent stem cell, Red blood cell

## Abstract

**Background:**

Blood transfusion is an essential part of medicine. However, many countries have been facing a national blood crisis. To address this ongoing blood shortage issue, there have been efforts to generate red blood cells (RBCs) in vitro, especially from human-induced pluripotent stem cells (hiPSCs). However, the best source of hiPSCs for this purpose is yet to be determined.

**Methods:**

In this study, hiPSCs were established from three different hematopoietic stem cell sources—peripheral blood (PB), cord blood (CB) and bone marrow (BM) aspirates (*n* = 3 for each source)—using episomal reprogramming vectors and differentiated into functional RBCs. Various time-course studies including immunofluorescence assay, quantitative real-time PCR, flow cytometry, karyotyping, morphological analysis, oxygen binding capacity analysis, and RNA sequencing were performed to examine and compare the characteristics of hiPSCs and hiPSC-differentiated erythroid cells.

**Results:**

hiPSC lines were established from each of the three sources and were found to be pluripotent and have comparable characteristics. All hiPSCs differentiated into erythroid cells, but there were discrepancies in differentiation and maturation efficiencies: CB-derived hiPSCs matured into erythroid cells the fastest while PB-derived hiPSCs required a longer time for maturation but showed the highest degree of reproducibility. BM-derived hiPSCs gave rise to diverse types of cells and exhibited poor differentiation efficiency. Nonetheless, erythroid cells differentiated from all hiPSC lines mainly expressed fetal and/or embryonic hemoglobin, indicating that primitive erythropoiesis occurred. Their oxygen equilibrium curves were all left-shifted.

**Conclusions:**

Collectively, both PB- and CB-derived hiPSCs were favorably reliable sources for the clinical production of RBCs in vitro, despite several challenges that need to be overcome. However, owing to the limited availability and the large amount of CB required to produce hiPSCs, and the results of this study, the advantages of using PB-derived hiPSCs for RBC production in vitro may outweigh those of using CB-derived hiPSCs. We believe that our findings will facilitate the selection of optimal hiPSC lines for RBC production in vitro in the near future.

**Supplementary Information:**

The online version contains supplementary material available at 10.1186/s13287-023-03305-8.

## Background

Blood transfusion is an essential medical procedure, with its applications ranging from elective and emergent surgeries to chronic disorders such as thalassemia and other forms of anemia [1]. The number of red blood cell (RBC) units obtained from healthy donors and used for transfusions varies widely by country. In South Korea, about 2.4 million people donated blood in 2021 [2], but the demand for blood still surpassed the supply. In fact, South Korea has faced blood supply shortage for years, largely because of population aging and low birth rate [3, 4]. Other factors include holiday periods during which less people donate blood and unpredictable virus outbreaks [1]. Since the beginning of the COVID-19 pandemic in 2019, there has been a severe global blood shortage. Thousands of Red Cross blood drives were cancelled in the United States of America, resulting in the US blood centers having only one to two days' worth of supply [5]. In South Korea, the blood supply was depleted to such an extent that some blood collection centers advised hospitals to delay elective procedures and restrict blood transfusions [6]. Additionally, while RBC transfusions have been relatively safe after adopting various donor screening methods, there remain risks of alloimmunization and transfusion-transmitted infections [7].

To address this imbalance in the supply and demand for blood and to mitigate transfusion-related risks, attempts have been made to generate RBCs in vitro. In the 1960s, artificial oxygen carriers, such as hemoglobin- and perfluorocarbon-based oxygen carriers, were extensively studied but were ultimately declared nonviable due to serious complications [8, 9]. Since then, efforts have shifted to stem cell-derived RBC production. CD34+ hematopoietic stem cells from cord blood (CB) and granulocyte-colony stimulating factor-mobilized peripheral blood (PB) were used to produce RBCs in vitro, but their clinical use was limited because of low efficiency [9]. In the 2000s, human embryonic stem cells became popular as a potentially limitless and immortal source of RBCs but were subject to ethical controversy and potential tumorigenicity [10–13]. After the discovery of the Yamanaka factors, Oct3/4, Sox2, Klf4, and c-Myc, that were used to induce pluripotency through somatic cell reprogramming [14], human-induced pluripotent stem cells (hiPSCs), have emerged as a promising option because of their potential to differentiate into hematopoietic lineages and generate viable RBCs in vitro.

Many laboratories around the world have generated RBCs from hiPSCs after the first reported differentiation of hiPSCs into erythrocytes using a suspension embryoid body (EB) in 2010 [15], but the widely used three-dimensional method for forming EBs is labor-intensive and expensive [16]. In addition, hiPSC-derived RBCs used for therapeutic purposes should be produced in a serum-free, feeder-free condition, and on a large scale. However, to date, no standardized method has been developed to achieve this [16–20].

Over the decades, cells from various sources have been used to generate hiPSCs that could further differentiate into RBCs in vitro [1, 21, 22], but the best source of hiPSCs is yet to be determined. It was reported that all hiPSCs, regardless of the cell of origin, showed terminal maturation into normoblasts and enucleated reticulocytes, but those derived from CD34+ hematopoietic stem cells had higher growth rates than those derived from other types of cells [22], indicating that hematopoietic stem cell sources would be a good option for generating RBCs in vitro.

PB, CB, and bone marrow (BM) aspirates are all sources of hematopoietic stem cells. However, they vary in cellular characteristics [23]. It is still unknown whether hiPSCs derived from these three sources and their subsequent differentiated erythroid cells have similar characteristics and differentiation efficiencies. To investigate this, in this study, we constructed hiPSC lines from PB, CB, and BM and differentiated them into erythroid cells in a serum-free, feeder-free medium without forming an EB. We then identified and compared the characteristics of hiPSCs established from the three different hematopoietic stem cell sources and hiPSC-derived erythroid cells using cell morphological analysis, flow cytometric analysis, immunofluorescence analysis, gene expression profiling, karyotyping, globin expression profiling, and oxygen-binding capacity analysis under the same conditions. To the best of our knowledge, this is the first study to characterize the erythroid differentiation and maturation efficiencies of hiPSC lines derived from different hematopoietic stem cell sources. Comparing the characteristics of these cells will provide valuable information to assess the best hiPSC source for clinical applications and improve the differentiation process. Owing to the different cellular characteristics of the three different hematopoietic stem cell sources, and because some form of epigenetic memory of their tissue of origin is probably retained within the cells [24], we hypothesized that one source would be superior to the others and consequently, be the most suitable as a starting material of hiPSCs for RBC production in vitro.

## Methods

### Materials

The materials used for cell cultures and characterization are listed in Additional file 1: Table S1.

### Cell sources

After getting informed consent, PB was drawn from three healthy O, Rh D-positive donors. CB was collected from three healthy newborn babies at the Department of Obstetrics and Gynecology at Severance Hospital in Seoul, South Korea. Three normal frozen BM aspirate samples were acquired from the Department of Internal Medicine at the same hospital. The study protocol was reviewed and approved by the Institutional Review Board of Severance Hospital (IRB No. 4-2018-0890).

### Production of hiPSCs

#### Isolation of mononuclear cells

About 15–20 mL PB and BM aspirates and 100 mL umbilical CB were collected in a tube containing sodium heparin anticoagulant (BD Biosciences, Oxford, UK). Mononuclear cells (MNCs) were purified using 10 mL Ficoll-Paque (GE Healthcare, Uppsala, Sweden). Viable MNCs were counted using the trypan blue exclusion method [25].

#### Erythroid progenitor expansion

Viable MNCs were resuspended and plated at a density of 1 × 10^6^ cells/mL on erythroid expansion media composed of basal medium and erythroid cytokines. The composition of the basal medium was 150 μg/mL transferrin, 50 μg/mL insulin, 90 ng/mL ferrous nitrate, and 160 μM monothioglycerol in Stemline II media (all from Sigma-Aldrich, Gillingham, UK). The erythroid expansion medium was prepared by adding 10 μg/mL hydrocortisone (HC) (Sigma-Aldrich), 10 μg/mL stem cell factor (SCF) (Sigma-Aldrich), 10 μg/mL erythropoietin (EPO) (Stem Cell Technologies, Vancouver, Canada), and 1 μg/mL interleukin-3 (IL-3) (Peprotech EC Ltd., London, UK) to the basal medium. MNCs were suspended at a density of 1 × 10^7^ cells/mL in 10 mL erythroid expansion medium in a 25T flask (Thermo Fisher Scientific, Waltham, MA, USA) for three days in a 37 °C incubator in a humidified environment containing 5% CO_2_. On day 3, both adherent and non-adherent cells were collected into a 15-mL conical tube and centrifuged at 400× *g* for 5 min. The pellet was resuspended at a density of 1 × 10^6^ cells/mL in fresh erythroid expansion medium. Starting on day 5, microscopic morphological analysis was performed every day until the population of erythroid progenitor cells accounted for approximately 80% of the MNCs, at which point the cells were ready for transfection [26].

#### Transfection

Prior to transfection, each well in 6-well Nunc™ Multidishes (Thermo Fisher Scientific) was coated with a mixture of 14.5 μL Matrigel Matrix (Stem Cell Technologies) and 985.5 μL Dulbecco's Modified Eagle Medium (DMEM)/F12 (1X) (Gibco, Life Technologies, Carlsbad, CA, USA) for 1 h at room temperature. A total of 1 × 10^6^ expanded erythroid cells were centrifuged at 400× *g* for 5 min and resuspended in 100 μL Opti-MEM media (Gibco); 2 μL the Epi Episomal Reprogramming Vectors, pCE-hOCT3/4 (OCT4), pCE-hSK (SOX2, KLF4), and pCE-hUL (L-Myc, Lin28); and 2 μL Epi p53 and EBNA vectors, pCE-mP53DD (mp53DD), and pCXB-EBNA1 (EBNA1) (Life Technologies, Frederick, MD, USA). Prepared cells were transferred to Electroporation Cuvettes (Nepa Gene Co., Ltd., Chiba, Japan) and loaded into a NEPA21 Super Electroporator (Nepa Gene Co., Ltd.). Transfection was performed as per the manufacturer’s instructions. The transfected cells were transferred to 6 mL of erythroid expansion medium, and 2 mL of the mixed product was plated on a 6-well plate pre-coated with Matrigel at a density of 3.3 × 10^5^ cells/well. On the second and fifth days after transfection, each well was supplemented with 1 mL erythroid expansion medium and 1 mL ReproTeSR (Stem Cell Technologies), respectively. Beginning on post-transfection day 7, complete medium changes were performed with 2 mL ReproTeSR every day, and the morphology of colonies was closely monitored until the hiPSC-like colonies appeared [26, 27].

#### Maintenance of hiPSCs

The produced hiPSCs were maintained on the plates with 2 mL mTeSR Plus Basal media (Stem Cell Technologies) in an incubator containing 5% CO_2_ at 37 °C and cultured daily with fresh mTeSR until the cells reached 80–90% confluence. Within five to seven days, hiPSC colonies were ready for passaging. For newly reprogrammed hiPSCs up to approximately passage five, colonies were manually passaged using a loop made from a heated glass Pasteur pipette to select only preferred colonies of undifferentiated hiPSCs. For the subsequent passages, hiPSCs were enzymatically passaged using ReLeSR (Stem Cell Technologies). The medium was changed daily. Cultured hiPSCs between passages 10 and 15 were used for erythroid differentiation in the study [26].

### Differentiation into hematopoietic stem cell and erythroid cell lineages

**Fig. 1:**
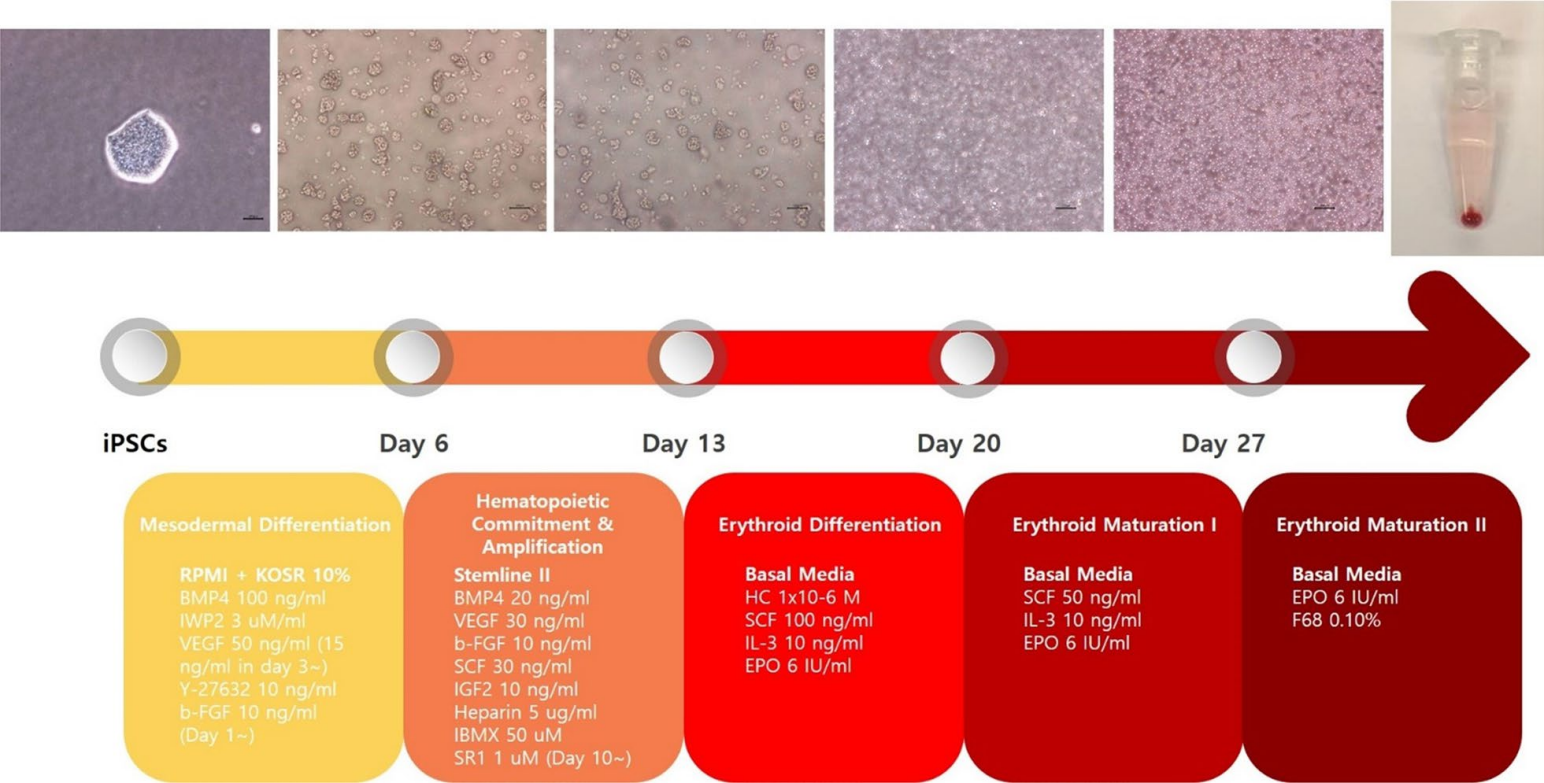
Diagrammatic representation of the hematopoietic and erythroid differentiation of hiPSCs. Abbreviations: KOSR, Knock Out Serum Replacement; BMP4, bone morphogenetic proteins 4; VEGF, vascular endothelial growth factor; b-FGF, fibroblast growth factor-basic; SCF, stem cell factor; IGF2, insulin-like growth factor 2; IBMX, 3-isobutyl-1-methylxanthine; SR1, StemReagenin1; HC, hydrocortisone; IL-3, interleukin-3; EPO, erythropoietin

We used a modified stepwise protocol cited in other studies [19, 26]. The diagram depicting the hematopoietic and erythroid differentiation of hiPSCs is illustrated in Fig. 1.

#### Mesodermal differentiation

On day 0 of differentiation, a new medium composed of Roswell Park Memorial Institute (RPMI) (Gibco) with 10% KnockOut Serum Replacement (KOSR) (Gibco), 100 ng/mL bone morphogenetic proteins 4 (BMP4), 3 μM/mL IWP2, 50 ng/mL vascular endothelial growth factor (VEGF), and 10 ng/mL Y-27632 was prepared. The existing medium of hiPSC colonies was removed, and 2 mL Dulbecco’s phosphate-buffered saline (DPBS) was added to each well. After removing the DPBS, 1 mL ReLeSR was added and suctioned immediately. The plates were incubated in an incubator containing 5% CO_2_ for 3 min. Colonies of hiPSC were dissociated into small clumps containing 30–50 cells. After confirming the size of the clumps under a stereo microscope, the cells were resuspended in the prepared media at a density of 3–5 × 10^6^ cells/well on Costar 6-well Clear Flat Bottom Ultra-low Attachment Multiple Well Plates (Corning Life Sciences, Durham, NC, USA). On day 1, 10 ng/mL fibroblast growth factor-basic (b-FGF) was added to each well and maintained for one day. On day 3, a complete medium change using RPMI with 10% KOSR, 100 ng/mL BMP4, 3 μM/mL IWP2, 15 ng/mL VEGF, 10 ng/mL b-FGF, and 10 ng/mL Y-27632 was performed, and the cells were maintained for two more days. For complete medium changes, cell suspensions were centrifuged at 400× *g* for 5 min, and the cells were reseeded in fresh media.

#### Hematopoietic commitment and amplification

On day 6, half of the medium was exchanged for Stemline II hematopoietic stem cell expansion medium (Sigma-Aldrich), 20 ng/mL BMP4, 30 ng/mL VEGF, 10 ng/mL b-FGF, 30 ng/mL SCF, 10 ng/mL insulin-like growth factor 2 (IGF2), 5 μg/mL heparin, and 50 μM 3-isobutyl-1-methylxanthine (IBMX). On day 7, all cells were collected and centrifuged at 400× *g* for 5 min. The pellet was dissociated with medium containing Stemline II hematopoietic stem cell expansion medium (Sigma-Aldrich), 20 ng/mL BMP4, 30 ng/mL VEGF, 10 ng/mL b-FGF, 30 ng/mL SCF, 10 ng/mL IGF2, 5 μg/mL heparin, and 50 μM IBMX. The cells were re-plated in a 25T flask (Thermo Fisher Scientific) and maintained for three days. On day 10, a complete medium change was performed with the same freshly mixed medium used on day 7 with the addition of 1 μM StemReagenin1 (SR1), and the cells were cultured for three more days.

#### Erythroid differentiation and maturation

On day 13, the cells were re-plated in a basal medium with the following cytokines: 1 × 10^−6^ M, 100 ng/mL SCF, 10 ng/mL IL-3, and 6 IU/mL EPO. On day 20, a basal medium mixed with 50 ng/mL SCF, 10 ng/mL IL-3, and 6 IU/mL EPO was used. On days 13–27, a complete medium change was performed every three to four days. Starting on day 27, the cells were subjected to a complete medium change with basal media containing 6 IU/mL EPO and 0.10% F68 every three to four days until the cells fully matured into reticulocytes.

### Characterization of differentiated cells

#### Morphological analysis

Cells (1 × 10^5^ per slide) were centrifuged and immobilized onto a glass microscope slide using a Cytospin 5 cytocentrifuge (Thermo Fisher Scientific) at 700 rpm for 7 min. The slides were stained with Wright–Giemsa dye (Sigma-Aldrich), observed under a BX53 light microscope (Olympus, Tokyo, Japan), and imaged with a DP70 camera (Olympus) [26, 27].

#### Flow cytometric analysis

To investigate the expression of the hiPSC pluripotency markers, *SSEA4* and *TRA-1-60*, hiPSCs were dissociated using Gentle Cell Dissociation Reagent (Gibco) and centrifuged at 400× *g* for 5 min. The pellet was mixed with autoMACS Running Buffer (Miltenyi Biotec B.V. & Co. KG, Bergisch Gladbach, Germany) and aliquoted into cryotubes at a density of 1 × 10^5^ cells/200 μL with 0.5 M ethylenediaminetetraacetic acid (pH 8.0, 1:90 DPBS). Fluorochrome-conjugated antibodies (10 μL/10^5^ cells) were added to the cryotubes and incubated in the dark for 1 h at room temperature. Unbound antibodies were washed with 1000 μL Fluorescence-Activated Cell Sorting (FACS) buffer. After centrifugation at 400× *g* for 5 min, the pellet was resuspended in 500 μL 4% paraformaldehyde (Tech&Innovation, Gyeonggi-do, South Korea) for fixation.

To evaluate the hematopoietic and erythroid characteristics of differentiated cells on days 0, 13, 20, 27, 31, and 34, flow cytometric analysis was performed using antibodies against CD34-PE, CD43-APC, CD235a-PE, and CD71-APC (all from BD Biosciences, Oxford, UK). Cells were centrifuged at 400× *g* for 5 min, and the pellet was dissolved in 400 μL FACS buffer. The subsequent steps were the same as those mentioned above. Non-specific immunoglobulin isotype controls of the corresponding class served as a negative control. Compensation beads were used to modify compensation matrices.

Stained samples were measured in a BD Verse flow cytometer (BD Biosciences). FlowJo software version 10.2 (LLC, Ashland, OR, USA) was used to analyze the data [26–28].

#### Immunofluorescence assay

Reprogrammed cells were gently washed with DPBS and fixed in 4% paraformaldehyde at room temperature for 20 min. The cells were washed again with 0.05% Tween-20 (Sigma-Aldrich) and permeabilized with 0.1% Triton X-100 (Sigma-Aldrich) at room temperature for 15 min. After washing, they were blocked with 4% Donkey Serum at 4 °C overnight and wrapped in parafilm. Primary antibodies, SSEA4, OCT4, SOX2, TRA-1-60, and NANOG, were added to the cells and then, incubated at 4 °C overnight. The secondary antibodies, Alexa Fluor 594 anti-rabbit or Alexa Fluor 488 anti-rabbit antibodies (Life Technologies, Eugene, OR, USA), were supplemented after washing with 0.05% Tween-20. The samples were incubated at 4 °C overnight in the dark and stained with UltraCruz Aqueous Mounting Medium with DAPI (Santa Cruz Biotechnology, Dallas, TX, USA) for observation.

A CKX53 fluorescence microscope (Olympus, Tokyo, Japan) with a U-RFL-T fluorescence lamp (Olympus) was used to visualize the cells. Image analysis and colocalization studies were carried out using the Ocular Image Acquisition Software, version 2.0.1.496 (Digital Optics Limited, Auckland, New Zealand) [26].

#### Karyotyping

hiPSCs were fixed and examined using standard G-banding analysis for karyotyping, which was performed by GenDX (Seoul, South Korea) utilizing a GTG-banding technique [29].

#### Quantitative real-time polymerase chain reaction (qRT-PCR) for detection of various markers

For pluripotent stem cell markers, hiPSCs were grown for four to five days and cultured for analysis. For the three germ layer markers, hiPSCs were maintained on a 10-cm plate coated with 0.1% gelatin solution (GenDEPOT, Katy, TX, USA) in 10 mL germ layer differentiation media composed of DMEM (Gibco) and 10% Fetal Bovine Serum (Gibco). The cells were grown for two weeks with complete medium changes every two to three days.

Total RNA from hiPSC samples was extracted using the RNeasy Plus Mini Kit (Qiagen, Hilden, Germany). Complementary DNA was generated using the iScript cDNA Synthesis Kit (BIO-RAD, Hercules, CA, USA). qRT-PCR was performed using TaqMan Gene Expression Master Mix (Applied Biosystems, Foster City, CA, USA) and analyzed using the Step One Plus (Applied Biosystems). The glyceraldehyde 3-phosphate dehydrogenase (*GAPDH*) gene was used to normalize data, and relative expression was calculated using the ΔΔCT method. The statistical significance of the differences between samples was analyzed using a two-way analysis of variance (ANOVA) in GraphPad Prism 9, version 9.2.0 (GraphPad Software, San Diego, CA, USA).

The following qPCR TaqMan probes (Applied Biosystems) were used: POU5F1(OCT4) Hs04260367_gH_FAM; NANOG H02387400_g1_FAM; SOX2 Hs01053049_s1_FAM; KLF4 Hs00358836_m1_FAM; c-MYC Hs00153408_m1_FAM; Brachyury Hs00610080_m1_FAM; Sox17 Hs00751752_s1_FAM; Nestin Hs04187831_g1_FAM; and GAPDH Hs02758991_g1_VIC [21, 26, 30, 31].

#### Hemoglobin composition analysis using quantitative real-time PCR

On day 31, total RNA from hiPSC-derived erythroid cells was extracted using an RNeasy Plus Mini Kit (Qiagen). Complementary DNA was prepared using an iScript cDNA Synthesis Kit (BIO-RAD) and PowerSYBR Green PCR Master Mix (Life Technologies, Ltd., Warrington, UK). qRT-PCR was performed using Step One Plus (Thermo Fisher Scientific) as per the manufacturer's instructions. *GAPDH* was used as the house keeping gene for normalizing sample quantities. Relative expression was calculated using the ΔΔCT method. Primers were synthesized by Bioneer (Seoul, South Korea) (Additional file 1: Table S2) [16, 30].

#### Oxygen-binding capacity analysis

A Hemox-Analyzer (TCS, Southampton, PA, USA) was used to determine the oxyhemoglobin dissociation curves and *P*_50_ values of the samples on day 34. Five milliliters HEMOX solution, 20 μL Additive-A, and 10 μL anti-foaming agent (all from TCS) were added to the sample vial and mixed with the sample. The vial was loaded into the cuvette, and the analysis was performed as per the manufacturer’s instructions. The data obtained from hiPSC-differentiated erythroid cells were compared to those obtained from freshly drawn normal PB [21, 27, 32].

#### Scanning electron microscopy

On day 34, hiPSC-derived erythroid cells were fixed in Karnovsky’s fixative, which was composed of 2% glutaraldehyde and 2% paraformaldehyde in 0.1 M phosphate buffer at a pH of 7.4 (all from Merck, Darmstadt, Germany), for 24 h and washed with 0.1 M phosphate buffer. The prepared samples were post-fixed with 1% osmium tetroxide (Polysciences, Washington, PA, USA) for 2 h and dehydrated through incubation in a series of ethanol solutions (50–100%). Critical point-drying was performed using an EM CPD300 (Leica Microsystems, Wetzlar, Germany) for 1–2 h. The specimens were mounted using a Stemi 305 (Zeiss, Jena, Germany) and coated with platinum using a Leica EM ACE600 ion sputtering system (Leica Microsystems, Wetzlar, Germany). The prepared specimens were observed using a Merlin field emission electron microscope (Zeiss, Jena, Germany) [33].

#### RNA sequencing

For transcriptomic comparison of hiPSCs, RNA sequencing was carried out on six hiPSC lines (*n* = 2 for each source), and the genes expressed in CB- and BM-derived hiPSCs were compared to those obtained from PB-derived hiPSCs, which were set as control. To identify changes in transcriptomic expression during differentiation, RNA sequencing was performed on days 13, 20, and 27 (*n* = 1 for each source), corresponding to three stages of erythroid differentiation, and the transcriptomic profiles on each day were compared to those of their precursor hiPSCs.

Samples of approximately 1 × 10^7^ cells/mL on days 0, 13, 20, and 27 were lysed using 1 mL TRIzol Reagent (Life Technologies) according to the manufacturer’s instructions and stored at − 70 °C until RNA extraction. RNA preparation, sequencing, and analysis were all performed by Macrogen, Inc. (Seoul, South Korea). The quality of isolated RNA samples was evaluated with Tape Station RNA Screen Tape (Agilent, Santa Clara, CA, USA). cDNA libraries were prepared using a TruSeq Stranded mRNA LT sample Prep Kit (Illumina, San Diego, CA, USA), and the transcribed cDNA was sequenced using a NovaSeq 6000 (Illumina).

The raw sequences were quality-checked using FastQC v.0.11.7, and low-quality bases and the adaptor contamination were removed using Trimmomatic v.0.38. Trimmed reads were mapped to the human genome reference, namely either UCSC hg19 or GRCh37, using HISAT2 (Johns Hopkins University, Baltimore, MD, USA). The total mapped read numbers were determined and normalized to detect the number of fragments per kilobase of transcript per million mapped reads and transcripts per kilobase. Differentially expressed gene analysis was performed using these values [34, 35].

### Statistics and reproducibility

At least three independent differentiation experiments were performed for each cell line. All statistics were analyzed using GraphPad Prism, version 9.2.0 (GraphPad Software, San Diego, CA, USA). Values were considered statistically significant if the p-value was less than 0.05.

## Results

### hiPSCs were successfully established from all three sources and shared similar characteristics

**Fig. 2:**
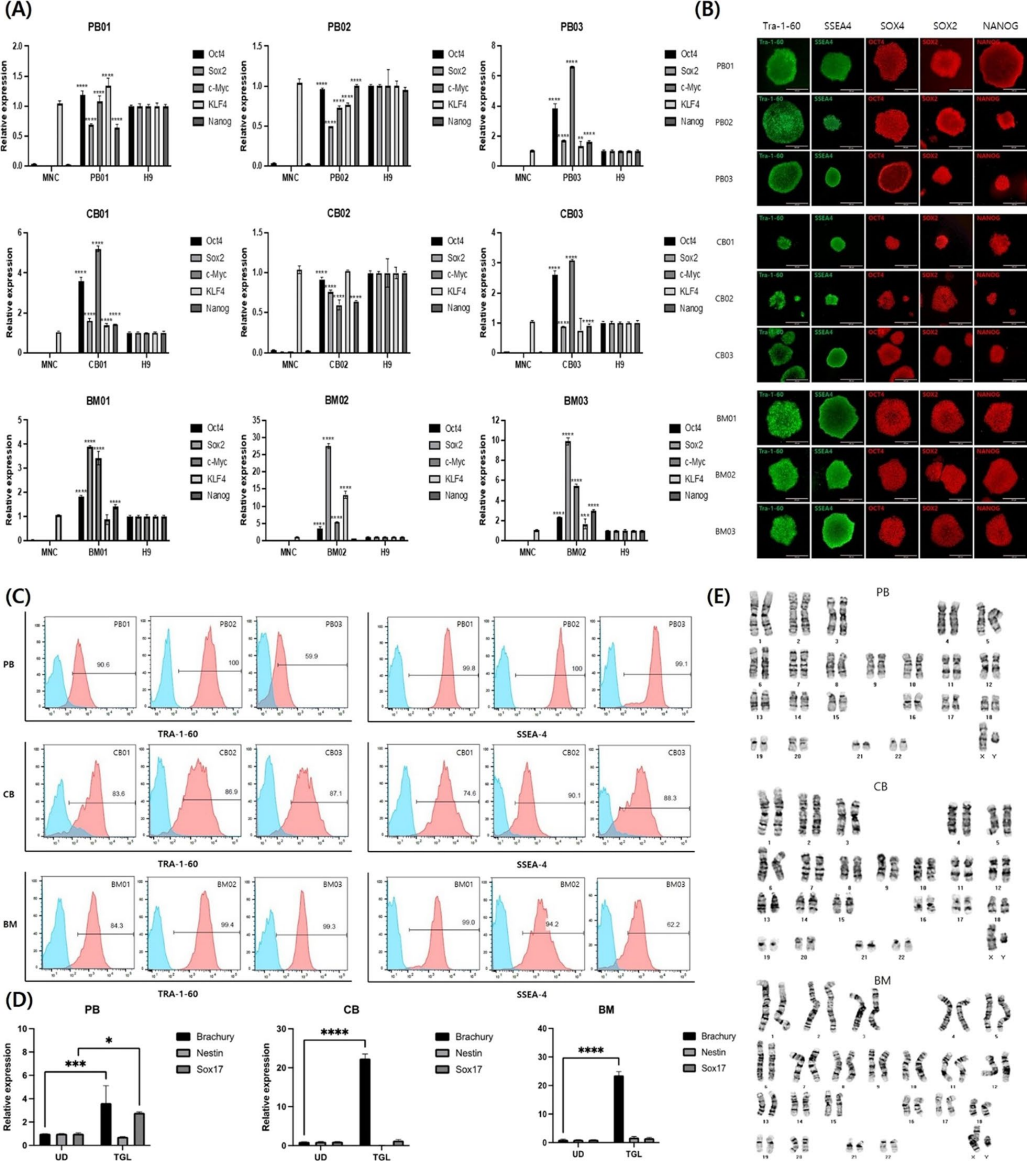
Characterization of human-induced pluripotent stem cells (hiPSCs) derived from three different sources. **A** Quantitative real-time polymerase chain reaction (qRT-PCR) results of the reprogramming factors *OCT4*, *SOX2*, *c-MYC*, *KLF4*, and *NANOG*. **B** Immunofluorescence assay results of the pluripotency markers, TRA1-60, SSEA4, SOX4, SOX2, and NANOG. **C** Flow cytometric analysis results of *TRA-1-60* and *SSEA4*. **D** qRT-PCR results of the three germ layer markers, Brachyury (mesoderm), SOX17 (endoderm), and Nestin (ectoderm). **E** Karyotyping results. Abbreviations: PB, peripheral blood; CB, cord blood; BM, bone marrow

Using episomal reprogramming factors, hiPSCs were successfully established from PB, CB, and BM. Cells were transfected when erythroblasts accounted for more than 80% of the population, which usually occurred on day 7 (Additional file 1: Fig. S1). Despite individual variations, hiPSC colonies appeared between days 14 and 24 after transfection with a yield of 1–20 colonies per 1 × 10^6^ MNCs. Among the three sources, CB exhibited the highest reprogramming efficiency (data not shown).qRT-PCR, immunofluorescence staining, flow cytometry, and chromosomal analysis were conducted to characterize the established hiPSCs (Fig. 2). qRT-PCR revealed the expression of the five transfected reprogramming factor genes, *OCT4*, *SOX2*, *c-MYC*, *KLF4*, and *NANOG* (Fig. 2A), and three germ layer markers, Brachyury (mesoderm), SOX17 (endoderm), and Nestin (ectoderm) (Fig. 2D). Immunofluorescence staining of the pluripotency markers, OCT4, SOX2, NANOG, TRA-1-60, and SSEA4, showed that clones of hiPSCs retained the typical characteristics of pluripotent stem cells, validating the pluripotency of all hiPSC lines (Fig. 2B). Flow cytometry also showed that cells expressed TRA-1-60 and SSEA4 (Fig. 2C). Chromosomal analysis revealed normal karyotypes for the hiPSCs (Fig. 2E). Furthermore, transcriptomic comparison of hiPSCs revealed that the expression levels of several selected genes that play an important role in erythropoiesis, such as *GATA1*, *KLF1*, *TAL1*, *c-KIT*, and *GFI1B*, were not statistically different between hiPSCs derived from different cellular sources (Additional file 1: Fig. S2A). Thus, all hiPSC lines, regardless of the cellular origins, were pluripotent and shared similar characteristics.

### hiPSCs from different sources showed successful differentiation into erythroid cells but with varied differentiation rates and efficiency

We used a modified stepwise protocol to determine whether hiPSCs could differentiate into erythroid cells. With differences in fold expansion of cells that were not statistically significant in each line during differentiation (data not shown), we achieved up to 7.3 × 10^7^ cells/mL within 34 days of differentiation. The characteristics of the differentiated cells were evaluated at different points in time for comparison as follows.

**Fig. 3:**
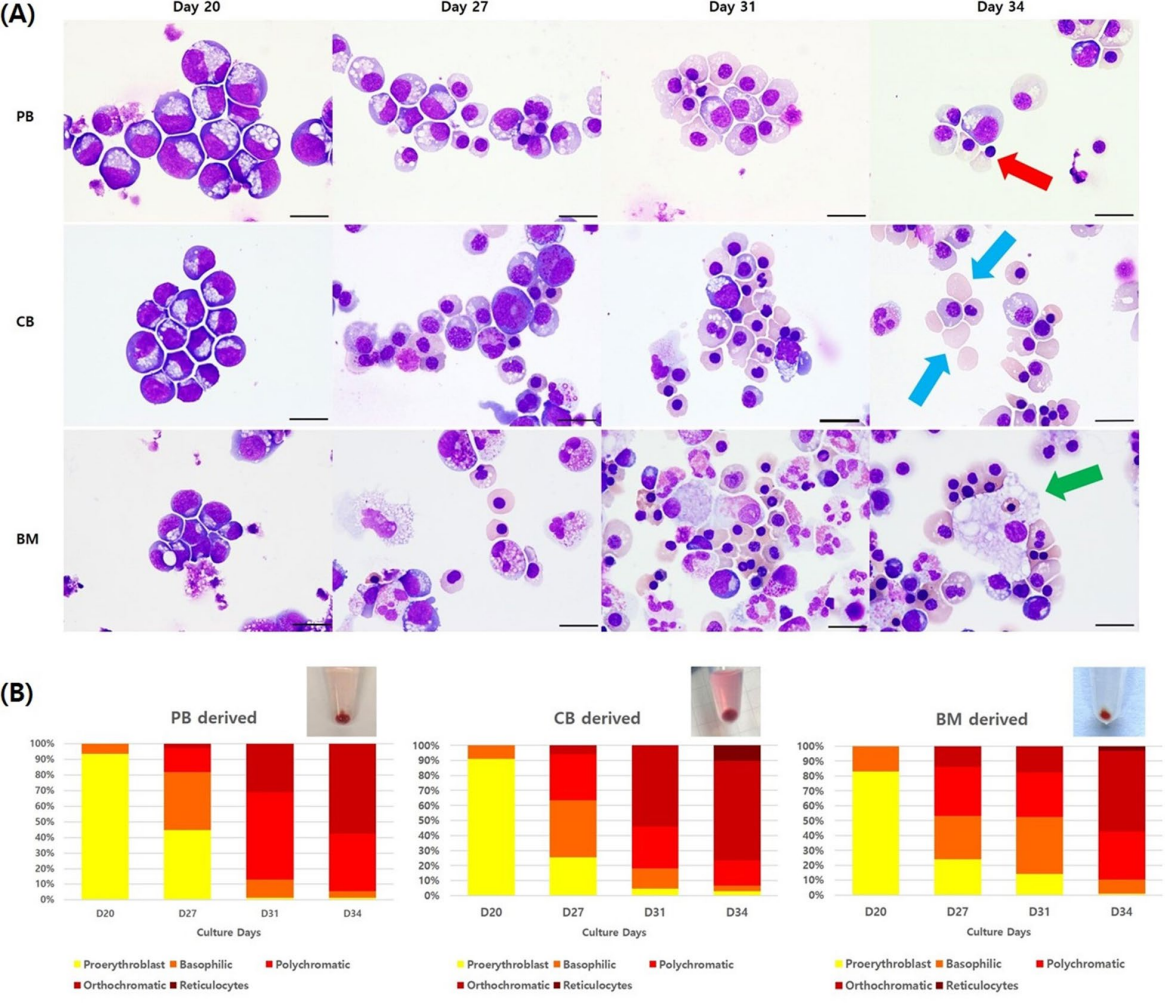
Morphological analysis results and differential counts of hiPSC-differentiated cells during differentiation. **A** Wright–Giemsa staining images of enucleating reticulocytes with an extruded nucleus (red arrow), enucleated reticulocytes (blue arrows), and histiocytes (green arrow) observed in hiPSC-differentiated erythroid cells over time (1000× magnification, scale bar = 10 μm). **B** Differential counts of erythroid cell subpopulation ratios during differentiation. Abbreviations: PB, peripheral blood; CB, cord blood; BM, bone marrow

First, cytospin slides stained with Wright–Giemsa were examined to determine the morphology of differentiated cells on days 20, 27, 31, and 34 (Fig. 3A). Despite some differences within and between hiPSC lines, all showed a shift in the population over time from hematopoietic stem cells to more mature forms of erythroid cells. In general, most of the cells observed on day 20 from all three sources were proerythroblasts with a small number of basophilic erythroblasts. Starting on day 27, polychromatic and orthochromatic erythroblasts became noticeable and accounted for the majority of cells on day 31. However, marked differences were detected on the final day of differentiation, day 34, where the number of enucleated reticulocytes in the CB-derived hiPSC group was greater than that in the other two groups, and several enucleating reticulocytes with extruded nuclei were observed in PB-derived hiPSC group. Moreover, BM-derived hiPSCs gave rise to other types of hematopoietic stem cell-derived lineages such as histiocytes, besides erythrocytes, when compared to the other hiPSC lines. Other lineage cells accounted for 26.3 ± 6.1% of cells in BM-derived samples compared to 6.5 ± 3.6% and 13.3 ± 5.5% in PB- and CB-derived samples, respectively. In addition, by counting the number of differentiated cells each day, CB-derived hiPSCs were shown to mature slightly faster than hiPSCs derived from the other two sources (Fig. 3B).

**Fig. 4:**
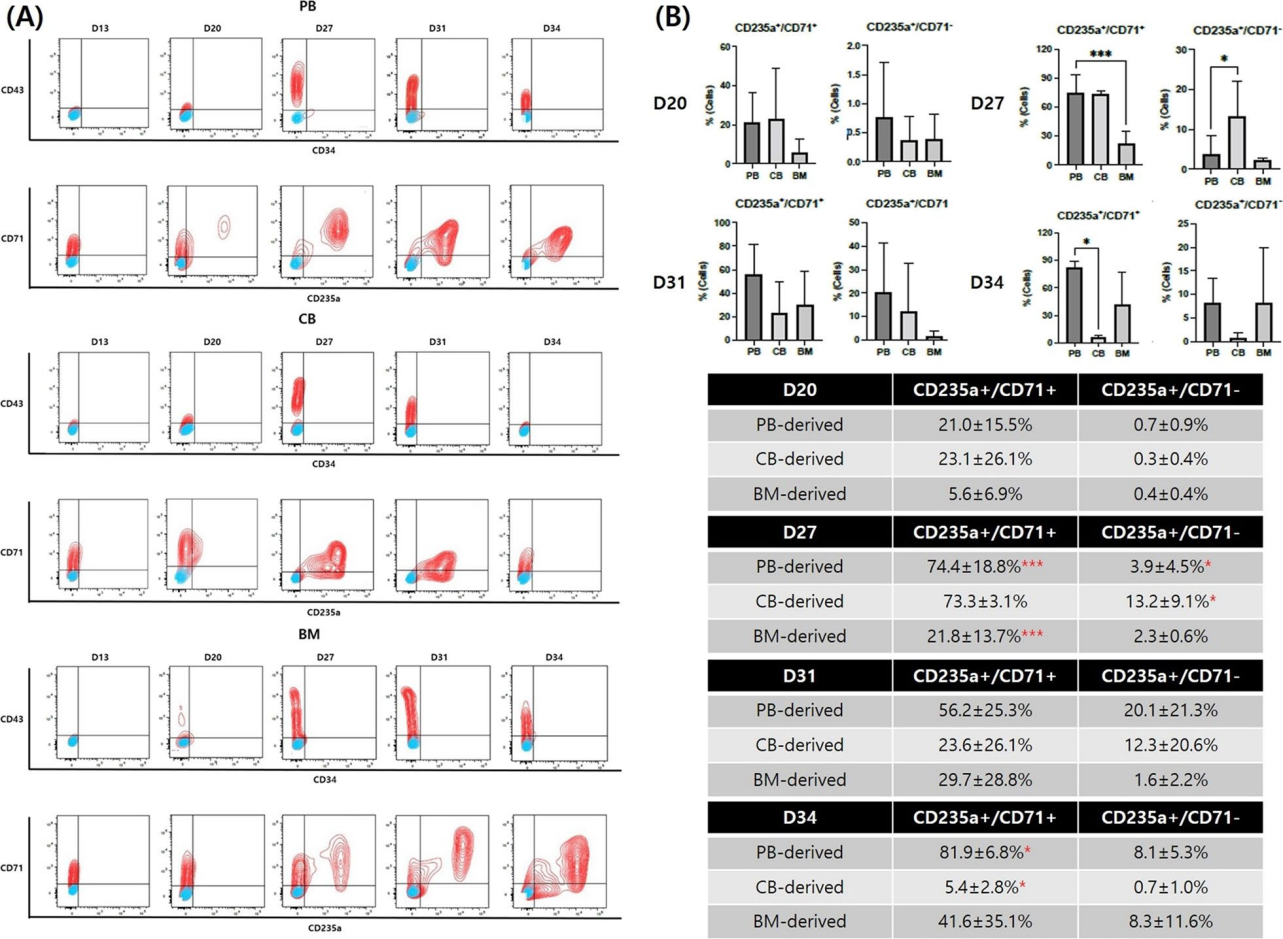
Flow cytometric analysis of hematopoietic and erythroid markers during differentiation. **A** Chronological shift of CD markers over time. **B** Comparison of CD235a+/CD71+ and CD235a+/CD71− cell counts during differentiation presented as mean ± SD [*p < 0.05, **p < 0.01, ***p < 0.001, ****p < 0.0001 (*n* = 3)]. Abbreviations: PB, peripheral blood; CB, cord blood; BM, bone marrow

Flow cytometry also showed that cell populations shifted from hematopoietic stem cells to more mature erythroid cells in all hiPSC lines over time (Fig. 4A). Noticeably, the hematopoietic marker, CD34, was weakly expressed or even absent during and after hematopoietic differentiation. Another hematopoiesis-specific marker, CD43, which usually appears in early hematopoietic progenitors and persists in differentiating precursor cells [22], started to appear on day 20. The percentage of cells expressing CD43 increased first and then decreased as differentiation progressed. The erythroid marker, CD235a, was first expressed between days 20 and 27, and its expression levels were consistent thereafter. The expression of the transferrin receptor, CD71, remained relatively high throughout the culture period but peaked on day 27 followed by a slight decrease, indicating the appearance of mature RBCs after day 27.

To compare the differentiation rate and efficiency, we compared the proportions of cells expressing CD235a+/CD71+, indicating immature RBCs, and CD235a+/CD71−, indicating mature RBCs, on days 20, 27, 31, and 34 between the three groups (Fig. 4B). On day 20, no significant difference was observed; erythroid differentiation occurred at similar times in all hiPSC lines. On day 27, however, the proportion of cells expressing CD235a+/CD71+ was significantly lower in BM-derived lines than that in PB-derived lines, reflecting poor erythropoietic status in the former. In addition, the percentage of cells expressing CD235a+/CD71− in CB-derived lines was significantly higher than that in PB-derived lines, indicating that terminal maturation occurred relatively early in CB-derived hiPSCs. The proportions of cells expressing CD235a+/CD71+ and CD235a+/CD71− in CB-derived hiPSCs were significantly and relatively low, respectively, on the final day of differentiation, day 34, reflecting early apoptosis compared to hiPSCs derived from other cell sources.

Transcriptomic analysis using RNA sequencing further supported the findings observed in morphological and flow cytometric analyses. Continuous upregulation of several key erythroid transcription factors, *GATA1*, *KLF1*, *TAL1*, *GFI1B*, and *STAT5*, reflected the shift from hematopoietic stem cells to mature erythroid cells and the success of erythropoiesis in all hiPSCs except slightly decreased expression of *KLF1* on day 27 in BM-derived line. (Additional file 1: Fig. S2B). It was also noted that the expression of *FLI1*, *FLT3*, and *ETV6*, which are non-erythroid transcription regulators [36, 37], was not downregulated as the differentiation progressed (Additional file 1: Fig. S2B). The expression of *GATA2* fluctuated and even peaked in the later stage of differentiation in both PB- and CB-derived lines (Additional file 1: Fig. S2B). In addition, globin gene expression levels on day 27 were significantly higher in erythroid cells differentiated from CB-derived hiPSCs and lower in those differentiated from BM-derived hiPSCs (Additional file 1: Fig. S2C) compared to those differentiated from PB-derived hiPSCs. This difference in globin gene expression levels reflected the difference in the maturation of hemoglobin-containing erythroid cells.

### All hiPSC lines showed continued erythropoiesis, even on the final day of differentiation, with low enucleation rates and revealed primitive erythropoiesis

**Fig. 5:**
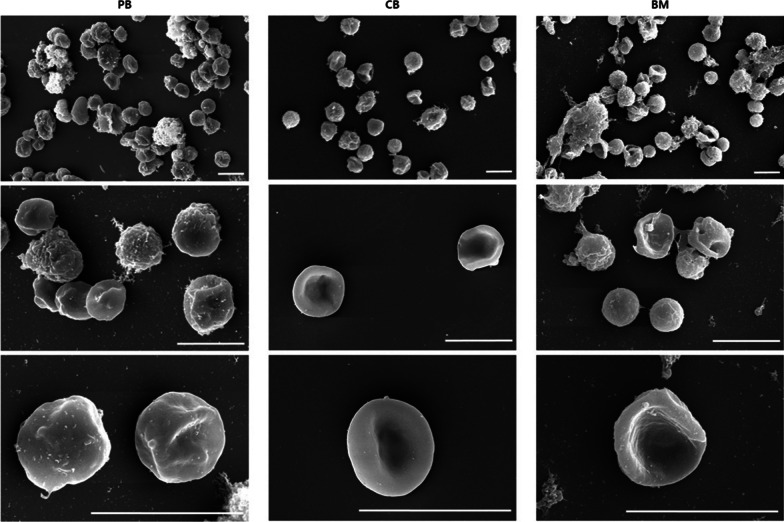
Scanning electron microscopy images of erythroid cells on day 34 (scale bar = 10 μm). Abbreviations: PB, peripheral blood; CB, cord blood; BM, bone marrow

Differential counting and flow cytometric analysis revealed that relatively high number of cells concurrently expressed CD235a and CD71 with low enucleation rates (≤ 10% in all hiPSC lines) on day 34, as shown in Figs. 3 and 4. This indicated that immature erythroid cells were still present and that the terminal erythropoiesis was not completed, even on the final day of differentiation. Scanning electron microscopy images taken on day 34 also showed that all hiPSCs gave rise to a heterogeneous population of cells with various phenotypes, including spherocytes, enucleating reticulocytes with extruded nuclei, and mature RBCs with a biconcave shape (Fig. 5), reflecting continued erythropoiesis in all hiPSC lines.

**Fig. 6:**
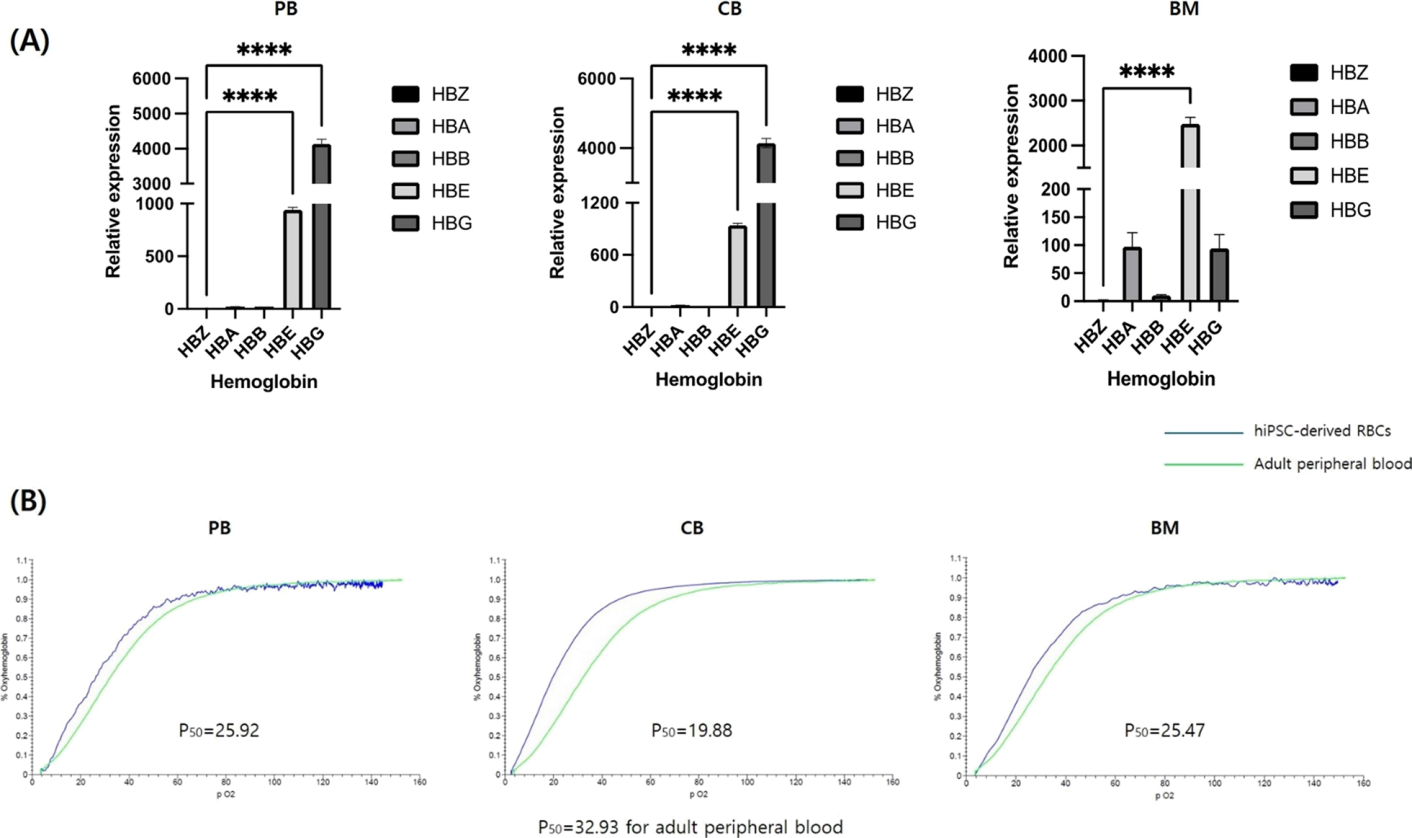
hiPSC-derived erythroid cells at the end of the differentiation process indicating primitive erythropoiesis. **A** Hemoglobin type qRT-PCR analysis results for erythroid cells on day 31 as determined using ANOVA [*p < 0.05, **p < 0.01, ***p < 0.001, ****p < 0.0001 (*n* = 3)]. **B** Oxygen equilibrium curves of erythroid cells on day 34. Abbreviations: ANOVA, analysis of variance; HB, hemoglobin; PB, peripheral blood; CB, cord blood; BM, bone marrow

On day 31, globin proportions in differentiated cells were examined using the qRT-PCR (Fig. 6A). Erythroid cells differentiated from PB- and CB-derived hiPSCs mainly expressed combinations of fetal and embryonic globin, whereas those differentiated from BM-derived hiPSCs mostly expressed embryonic globin along with some alpha and fetal globin. Although the composition of expressed globin slightly differed between cells derived from different hiPSC groups, all erythroid cells, regardless of the cellular origin, showed very low expression levels of adult globin compared to adult RBCs in PB, indicating that primitive, not definitive, erythropoiesis was predominant.

Oxygen equilibrium curves were also established to evaluate the oxygen-binding and dissociation capacities of the hemoglobin produced in hiPSC-derived erythroid cells on day 34 (Fig. 6B). Regardless of the hiPSC source, the curves were all left-shifted, and the *P*_50_ values of erythroid cells differentiated from PB- (*P*_50_ = 25.92), CB- (*P*_50_ = 19.88), and BM-derived hiPSCs (*P*_50_ = 25.47) were lower than those of RBCs in adult PB (*P*_50_ = 32.93). These findings were consistent with the dominant production of fetal and/or embryonic hemoglobin, which have a stronger affinity for oxygen compared to adult hemoglobin.

## Discussion

Due to the limited supply of blood products from healthy donors, a number of studies have attempted to generate RBCs in vitro [9]. Subsequently, hiPSCs were established as a potentially limitless source of blood, and many researchers have successfully differentiated hiPSCs, derived from various sources, into hematopoietic and erythroid cells for decades [1]. However, the most promising source of hiPSCs for producing RBCs in vitro is yet to be determined. Knowing that hiPSCs retain some form of epigenetic memory of their tissue of origin [24, 38], and that hiPSCs derived from hematopoietic stem cell sources show higher expansion rates and better final outcomes of the erythroid cells [22], we established hiPSCs from PB, CB, and BM, all being sources of hematopoietic stem cells with different cellular characteristics and contents [39]. We further differentiated these cells into erythroid cells using our robust, standardized method that included the expansion of MNCs, the generation and maintenance of hiPSCs, and the differentiation of hiPSCs into erythroid cells. During differentiation, we used various testing methods to determine how the characteristics of hiPSCs and hiPSC-differentiated erythroid cells from each source differed. We also assessed which source of hiPSCs would be the most reliable for RBC production in vitro. To minimize the number of variables that might affect cellular characteristics, we conducted all establishment and differentiation processes under the same conditions.

Our findings demonstrated that all three hematopoietic stem cell sources can be reprogrammed into hiPSCs with similar morphologies using episomal reprogramming vectors. However, CB-derived hiPSCs showed the highest reprogramming efficiency. This is consistent with the findings of a study by Raab et al. [40], which proposed that the reprogramming efficiency in CB group was high, whereas that in PB group was relatively low. Despite a difference in reprogramming efficiency, all hiPSC lines, regardless of the cellular origin, exhibited similar characteristics of pluripotency features and stable chromosomal integrity, as demonstrated in other studies [41]. Moreover, all cell lines were able to differentiate into erythroid cells but with distinctive differentiation and maturation efficiencies. Although all lines had comparable starting rates for erythroid differentiation, CB-derived hiPSCs matured the fastest but became apoptotic without differentiating into an acceptable number of enucleated cells. Cells differentiated from PB-derived hiPSCs mostly expressed CD235a/CD71 markers even on day 34, indicating the presence of several immature erythroid cells that needed more time to mature further. Nonetheless, PB-derived hiPSCs showed the highest degree of reproducibility. BM-derived hiPSCs produced various types of blood cells with a particularly high number of histiocytes and consistently lower expression levels of erythroid cell markers, demonstrating low differentiation efficiency.

Ever since the transcriptional and epigenetic patterns of murine iPSCs derived from different sources were first reported [42], many studies have shown an iPSC differentiation bias toward the lineage of the somatic cell of origin due to epigenetic memory [24, 41]. Contradictory findings that suggest epigenetic memory has minimal effects on differentiation have also been reported [43, 44]. As we did not have an opportunity to examine the epigenetic memory status of hiPSCs used in our study, we are unsure whether the discrepancies in differentiation rate and efficiency found in this study were due to the effect of epigenetic memory or other factors that need further investigation. Nevertheless, both PB- and CB-derived hiPSCs were promising sources for the production of RBCs in vitro, despite the many difficulties that still need to be overcome and are discussed later in this section. However, owing to the limited availability of donors, the large amount of CB needed to produce hiPSCs, and the relatively early apoptosis with low enucleation rates exhibited by CB-derived hiPSCs, the advantages of using PB-derived hiPSCs for RBC production in vitro may outweigh those of using CB-derived hiPSCs. Sivalingam et al. [21] focused on the suspension platform to generate high-density cultures of universal RBCs derived from hiPSCs, evaluated the differentiation of hiPSCs derived from various sources and selected the PB-derived hiPSC as the best-performing line for additional study, further supporting our findings.

As each unit of packed RBC consists of 2 × 10^12^ cells, cell densities of at least 1 × 10^8^ cells/mL are required to generate a suitable number of cells for transfusion [21]. Thus far, the fold increase for erythroid differentiation from hiPSCs varies according to the cell sources and differentiation methods used in previous studies, ranging from 10^3^ to 10^5^ fold with numerous variabilities [1], which is comparable with our results. Although the use of artificially produced RBCs in clinical setting is not immediately possible, determining the best source of hiPSCs is the first step to move forward. With the unlimited proliferation potential of hiPSCs, only 10 hiPSC clones are believed to be sufficient for nearly 99% of the alloimmunized patients [15].

RNA sequencing has become a common procedure for examining transcriptional profiles under different conditions; it has been widely employed in both academic and clinical settings [45]. In this study, we produced comprehensive transcriptome profiles of hiPSCs derived from three different hematopoietic stem cell sources and hiPSC-derived erythroid cells using RNA-sequencing at different points in time. A previous study compared the gene expression dynamics during erythropoiesis of hiPSCs to those of adult and CB progenitors [36]. To the best of our knowledge, however, this study is the first to compare gene expression profiles during erythropoiesis of hiPSCs derived from different hematopoietic stem cell sources. In normal BM, *GATA1* is expressed in all stages of differentiation and maturation [46], but its level peaks in the middle stages and declines during terminal erythroid differentiation [47]. However, the expression levels of *GATA1* in our study were consistently upregulated in all tested lines on day 27, indicating that differentiated erythroid cells were not fully mature yet. In addition, the fact that selected erythroid-related genes, such as *KLF1*, *TAL1*, *GFI1B*, and *STAT5*, were consistently upregulated while non-erythroid-related genes, such as *FLI1*, *FLT3*, and *ETV6*, were not completely suppressed during differentiation might support the hypothesis of incomplete erythropoiesis. Notably, the expression of *GATA2*, another important regulator of erythropoiesis, was high in the early stages but downregulated later during maturation [47] and peaked in the final stage of differentiation in all hiPSC lines in this study, probably because of differences between in vitro and in vivo conditions [46] that should be further explored.

All hiPSCs were differentiated into erythroid cells using our protocol; however, there were several limitations in our study pertaining to the clinical applicability of hiPSC-derived RBCs. We, and several other researchers, have shown that the relatively poor enucleation efficiency of hiPSC-derived erythroid cells is a critical obstacle to the generation of hiPSC-derived RBCs [1, 15, 21, 48]. Using small molecules or novel compounds [19, 21] and targeting various pathways that are known to be involved in the differentiation of erythroblasts into reticulocytes [1, 36, 49] could be explored further to overcome this complication. Yet another unsolved but potentially critical issue is the expression of fetal and/or embryonic hemoglobin and the control of its switching. Several studies, however, have reported that individuals with hereditary persistence of fetal hemoglobin are mostly asymptomatic and that the condition is primarily non-pathogenic [50, 51], and that this problem could be possibly solved by the activation of the globin switching genes, such as *KLF1* and *BCL11a* [52, 53], and elongating the hematopoietic differentiation periods [48]. Further studies on scaling up and producing robust amounts of RBCs are also necessary and could possibly be achieved using a bioreactor or through monitoring the metabolite level and replenishing depleted cytokines on a regular basis [21, 54].

We aim to conduct further studies on overcoming the limitations stated above to optimize the protocol and explore the transcriptomic changes during differentiation in vitro by increasing the sample sizes for RNA sequencing. This would help us understand the variations in the differentiation potential of hiPSCs derived from different sources and contribute to clinical applications.

## Conclusion

In this study, we used a stepwise protocol to generate erythroid cells from hiPSCs derived from three different hematopoietic stem cell sources, PB, CB, and BM, and compared the differentiation efficiencies and characteristics of the differentiated cells using different methods. We demonstrated that CB-derived hiPSCs showed the highest reprogramming and maturation speed with the fastest yet negligible rate of enucleation, whereas PB-derived hiPSCs exhibited delayed maturation but the highest reproducibility and accessibility, making them the most reliable source. BM-derived hiPSCs were an inadequate source for RBC production in vitro because of low erythropoiesis efficiency and reproducibility, but they could be used to produce other lineage cells. We believe that our findings will facilitate the selection of the optimal hiPSC lines for RBC production in vitro in the near future.

## Supplementary Information

**Additional file 1. Fig. S1:** Wright–Giemsa staining images of erythroblasts for transfection. Erythroblasts were counted every two to three days to determine the time of transfection. When the proportion of erythroid progenitor cells reached 80% or more of the cell population, the cells were considered ready for transfection. This threshold was usually reached on day seven of erythroblast expansion. (400 × magnification, scale bar = 20 μm). **Fig. S2:** Transcriptomic analysis of hiPSCs and hiPSC-differentiated erythroid cells. (**A**) Comparison of transcriptomes in hiPSCs derived from different sources. (**B**) Transcriptomic changes during differentiation. (**C**) Comparison of globin gene expression levels in hiPSC-differentiated erythroid cells on day 27. Abbreviations: PB, peripheral blood; CB, cord blood; BM, bone marrow; HB, hemoglobin. **Table S1:** List of materials used in this study. Table S2: Primers used for hemoglobin composition analysis. 

## Data Availability

All raw and processed sequencing data reported in this manuscript have been deposited in NCBI's Gene Expression Omnibus, and can be assessed through GEO series accession number GSE211625 at https://www.ncbi.nlm.nih.gov/geo/query/acc.cgi?acc=GSE211625.

## Abbreviations

ANOVA: Analysis of variance
b-FGF: Fibroblast growth factor-basic
BM: Bone marrow
BMP4: Bone morphogenetic proteins 4
CB: Cord blood
DMEM: Dulbecco’s modified Eagle medium
DPBS: Dulbecco’s phosphate-buffered saline
EB: Embryoid body
EPO: Erythropoietin
FACS: Fluorescence-activated cell sorting
GAPDH: Glyceraldehyde 3-phosphate dehydrogenase
HB: Hemoglobin
HC: Hydrocortisone
hiPSCs: Human-induced pluripotent stem cells
IBMX: 3-Isobutyl-1-methylxanthine
IGF2: Insulin-like growth factor 2
IL-3: Interleukin-3
KOSR: Knock out serum replacement
MNC: Mononuclear cells
PB: Peripheral blood
qRT-PCR: Quantitative real-time polymerase chain reaction
RBC: Red blood cell
RPMI: Roswell Park Memorial Institute
SCF: Stem cell factor
SR1: StemReagenin1
VEGF: Vascular endothelial growth factor
