# Supplementary material for: In vitro erythrocyte production using human-induced pluripotent stem cells: determining the best hematopoietic stem cell sources
Source: Stem Cell Res Ther. 2023 Apr 26;14:106. doi: 10.1186/s13287-023-03305-8 (PMC10132444; doi:10.1186/s13287-023-03305-8)
Supplement: Supplementary file 1 — Additional file 1. Fig. S1: Wright–Giemsa staining images of erythroblasts for transfection. Erythroblasts were counted every two to three days to determine the time of transfection. When the proportion of erythroid progenitor cells reached 80% or more of the cell population, the cells were considered ready for transfection. This threshold was usually reached on day seven of erythroblast expansion. (400 × magnification, scale bar = 20 μm). Fig. S2: Transcriptomic analysis of hiPSCs and hiPSC-differentiated erythroid cells. (A) Comparison of transcriptomes in hiPSCs derived from different sources. (B) Transcriptomic changes during differentiation. (C) Comparison of globin gene expression levels in hiPSC-differentiated erythroid cells on day 27. Abbreviations: PB, peripheral blood; CB, cord blood; BM, bone marrow; HB, hemoglobin. Table S1: List of materials used in this study. Table S2: Primers used for hemoglobin composition analysis. [file 13287_2023_3305_MOESM1_ESM.docx]

**Supplemental Information**


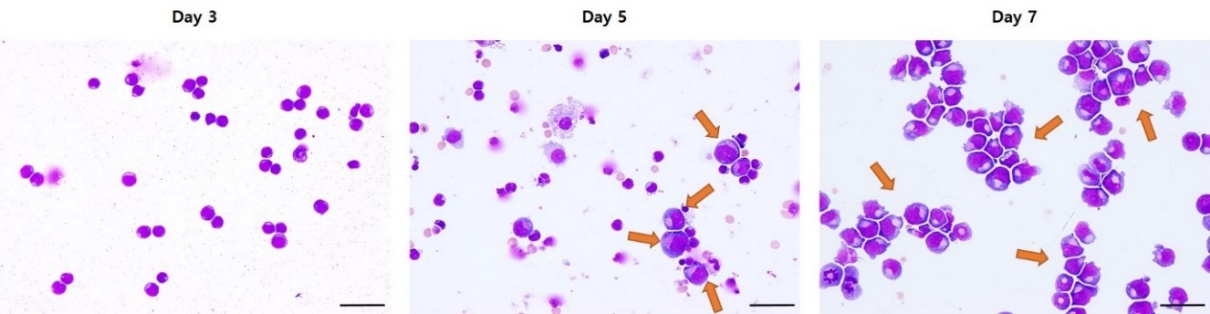


**Fig. S1.** Wright–Giemsa staining images of erythroblasts for transfection. Erythroblasts were counted every two to three days to determine the time of transfection. When the proportion of erythroid progenitor cells (orange arrows) reached 80% or more of the cell population, the cells were considered ready for transfection. This threshold was usually reached on day seven of erythroblast expansion. (400 × magnification, scale bar = 20 μm)

**
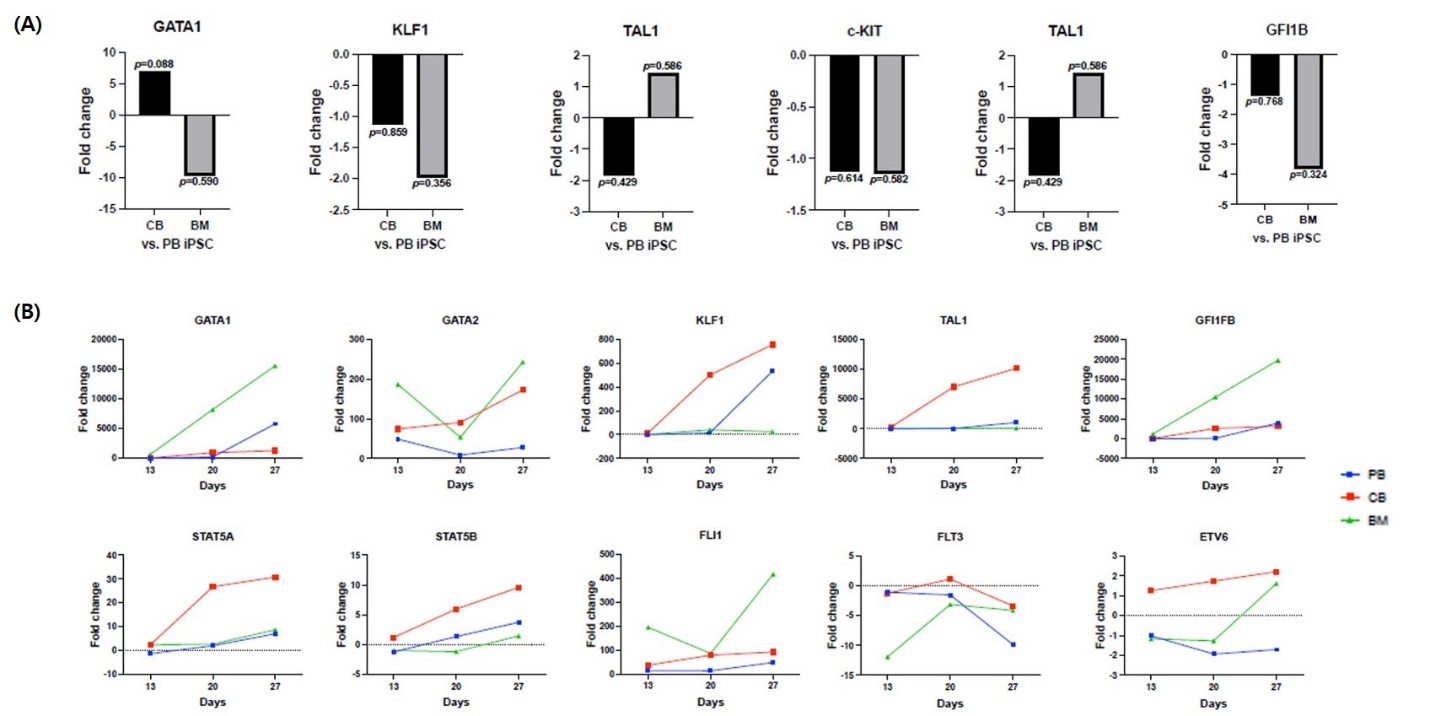
**

**
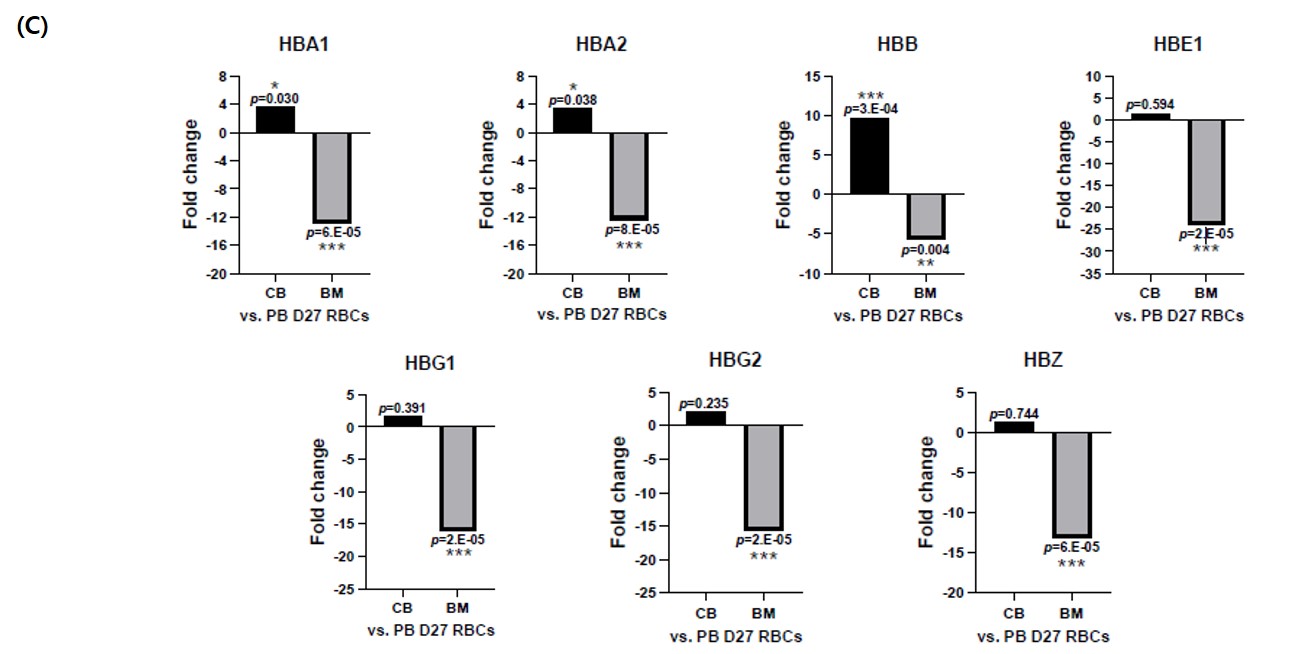
**

**Fig. S2.** Transcriptomic analysis of hiPSCs and hiPSC-differentiated erythroid cells. (A) Comparison of transcriptomes in hiPSCs derived from different sources. (B) Transcriptomic changes during differentiation. (C) Comparison of globin gene expression levels in hiPSC-differentiated erythroid cells on day 27. Abbreviations: PB, peripheral blood; CB, cord blood; BM, bone marrow; HB, hemoglobin.

**Table S1.** List of materials used in this study

| Materials | Abbreviations | Company | Catalogue number |
| --- | --- | --- | --- |
| Bone morphogenetic proteins | BMP4 | Peprotech | 120-05 |
| IWP-2 |  | Biogems | 6866167 |
| Vascular endothelial growth factor | VEGF | Peprotech | 100-20 |
| Fibroblast growth factor-basic | b-FGF | Peprotech | 100-18B |
| Y-27632 |  | StemCell Technologies | 72392 |
| Stem cell factor | SCF | Peprotech | 300-07 |
| Insulin-like growth factor 2 | IGF-2 | Peprotect | 100-12 |
| 3-Isobutyl-1-methylxanthine | IBMX | Sigma-Aldrich | I5879 |
| StemReagenin 1 | SR1 | Peprotech | 122499 |
| Hydrocortisone | HC | Sigma-Aldrich | H0888 |
| Interleukin 3 | IL-3 | Peprotech | 200-03 |
| Erythropoietin | EPO | Kyowa Kirin Korea |  |
| 10% Pluronic F-68 |  | Gibco | 24040032 |
| Transferrin | TF | Sigma-Aldrich | T8158 |
| Insulin |  | Sigma-Aldrich | I3536 |
| Ferric nitrate | FN | Sigma-Aldrich | F8508 |
| Monothioglycerol | MTG | Sigma-Aldrich | M6145 |
| Dulbecco's phosphate-buffered saline | DPBS | Gibco | 14190144 |
| RPMI 1640 medium |  | Gibco | A10491 |
| KnockOut Serum Replacement | KOSR | Gibco | 10828010 |
| Stemline II hematopoietic stem cell expansion medium |  | Sigma-Aldrich | S0192 |
| ReproTeSR |  | StemCell Technologies | 05920 |
| mTeSR1 Plus Basal Medium |  | StemCell Technologies | 100-0276 |
| ReLeSR |  | StemCell Technologies | 05872 |
| Nunc Cell-Culture Treated Multidish |  | Thermo Fisher Scientific | 140675 |
| Nunc EasYFlask 25cm^2^ |  | Thermo Fisher Scientific | 156367 |
| Costar 6-well Clear Flat Bottom Ultra-low Attachment Multiple Well Plates |  | Corning Life Sciences | 3471 |

**Antibodies used for flow cytometry**

| **Marker type** | **Antibody** | **Company** | **Catalogue number** |
| --- | --- | --- | --- |
| Embryonic and induced pluripotent stem cell markers | PE mouse anti-human TRA-1-60 | BD Biosciences | 560193 |
|  | Alexa Fluor 647 Mouse anti-SSEA4 | BD Biosciences | 560796 |
| Differentiation markers | PE Mouse Anti-Human CD34 | BD Biosciences | 555822 |
|  | APC Mouse Anti-Human CD43 | BD Biosciences | 560198 |
|  | PE Mouse Anti-human CD235a | Thermo Fisher Scientific | 12998782 |
|  | APC Mouse Anti-Human CD71 | BD Biosciences | 551374 |
| Isotype control | PE Mouse IgM | BD Biosciences | 555584 |
|  | APC Mouse IgG | BD Biosciences | 555751 |
|  | Alexa Fluor 647 IgG Mouse Isotype control | BD Biosciences | 557783 |

**Antibodies used for immunofluorescence assay**

| **Marker type** | **Antibody** | **Company** | **Catalogue number** |  |
| --- | --- | --- | --- | --- |
| **Primary**  **antibodies** | Rabbit anti-human OCT4 | Abcam | ab109884 |  |
|  | Rabbit anti-human SOX2 |  |  |  |
|  | Rabbit anti-human NANOG |  |  | |
|  | Mouse anti-human TRA-1-60 |  |  |  |
|  | Mouse anti-human SSEA4 |  |  |  |
| **Secondary antibodies** | Alexa Fluor 488 goat anti-mouse IgG | Invitrogen | A11001 |  |
|  | Alexa Fluor 594 donkey anti-rabbit IgG |  | A21207 |  |

| **Gene** | **Forward primer (5′🡪3′)** | **Reverse primer (3′🡪5′)** |
| --- | --- | --- |
| Hemoglobin alpha 1  (*HBA*) | CGGTCAACTTCAAGCTCCTAAG | CCGCCCACTCAGACTTTATT |
| Hemoglobin beta  (*HBB*) | TGGCCTGGCTCACCTGGACAA | GCGAGCTTAGTGATACTTGTGGGC |
| Hemoglobin epsilon 1 (*HBE*) | ATGGACAACCTCAAGCCCGCC | GGCAATGGCGACAGCAGACA |
| Hemoglobin gamma  (*HBG*) | CTTCAAGCTCCTGGGAAATGT | GCAGAATAAAGCCTATCCTTGAAAG |
| Hemoglobin zeta  (*HBZ*) | GATCTCCACGCAGGCCGACAC | GGTCCACGCGCAGGATGTAGG |
| Glyceraldehyde  3-phosphate dehydrogenase (*GAPDH*) | TGCACCACCAACTGCTTAGC | GGCATGGACTGTGGTCATGAG |

**Table S2.** Primers used for hemoglobin composition analysis [16]
